# Facile Fabrication of Highly Fluorescent N-Doped Carbon Quantum Dots Using an Ultrasonic-Assisted Hydrothermal Method: Optical Properties and Cell Imaging

**DOI:** 10.1021/acsomega.1c04903

**Published:** 2021-11-22

**Authors:** Chong Qi, Huaidong Wang, Ailing Yang, Xiaoxu Wang, Jie Xu

**Affiliations:** † College of Physics & Optoelectronic Engineering, Ocean University of China, Qingdao 266100, Shandong Province, China; ‡ College of Food Science and Engineering, Ocean University of China, Qingdao 266033, Shandong Province, China

## Abstract

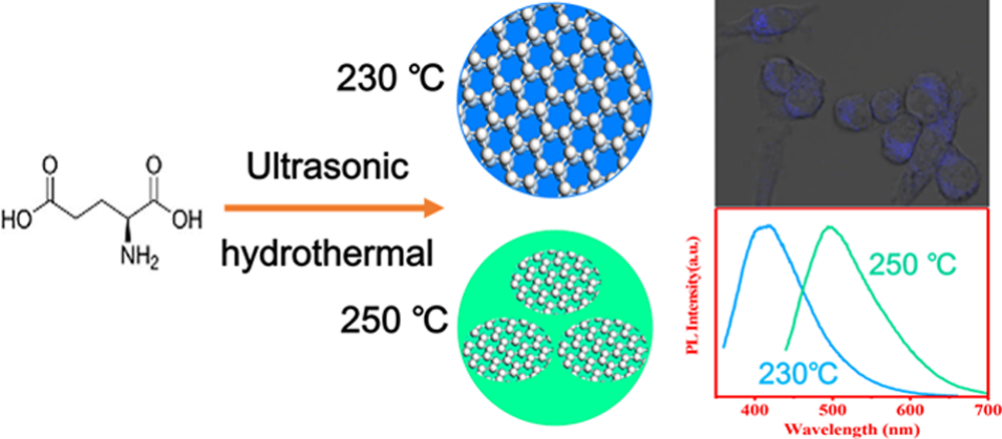

Fluorescent N-doped carbon nanodots (CNDs) are a type of environmentally friendly nanomaterial that is promising for application in cell imaging and optoelectronics. In this paper, a natural amino acid (l-glutamic acid) was used as a precursor, and two different morphological and structured N-doped carbon quantum dots (CQDs) were synthesized via a one-step ultrasonic-assisted hydrothermal method at 230 and 250 °C. Various microscopy and spectroscopy techniques were employed to characterize the morphology, structure, optical properties, and stability of the CQDs. The results showed that N-CQDs-1 are new CNDs composed of amorphous carbon with a large amount of pyroglutamic acid, and N-CQDs-2 are composed of pure amorphous carbon. The CQDs exhibit excellent optical properties, such as 40.5% quantum yield, strong photobleaching resistance, and superior photostability. Combining the fluorescence lifetimes and radiative and non-radiative decay constants, the photoluminescence mechanism of the CQDs was qualitatively explained. The two CQDs were used for BV2 cell imaging and showed good results, implying the ultrasonic-assisted hydrothermal approach as a facile method to obtain structure- and morphology-controllable N-doped CQDs with prospect for application in cell imaging.

## 1 Introduction

Quantum dots (QDs), a kind of semiconductor nanomaterial with a size smaller than the exciton Bohr radius, have attracted much attention in the last 30 years due to their unique surface effect, small size effect, and excellent electronic, optical, and electrochemical properties.^1^ QDs can be used in many fields such as light-emitting diodes (LEDs), artificial photosynthesis, biomedical imaging, and biosensing because of their high fluorescent quantum yield (QY), good photostability, and excellent photobleaching resistance.^2−4^ However, most of the high-performance QDs are composed of heavy-metal elements (i.e., Cd, Pb, and Hg),^5−9^ whose toxicities and potential environmental hazards limit their applications. To solve this problem, environment-friendly carbon-based fluorescent nanomaterials have aroused great research interests, especially carbon dots (CDs),^10−14^ a kind of zero-dimensional nanomaterials with a size less than 10 nm, which were first discovered in 2004.^15−17^ According to different carbon cores, CDs are usually divided into graphene quantum dots (GQDs), carbon nanodots (CNDs), and polymer dots.^18^ CNDs are divided into carbon nanoparticles without a crystal lattice and carbon quantum dots (CQDs) with an obvious crystal lattice. Irrespective of the types of CDs, they all have very small particle sizes and large specific surface areas. Their surface atoms are highly reactive and easily combine with other atoms or chemical groups to achieve different functions.^19^ Because of their merits in terms of low toxicity, strong photoluminescence (PL), good photostability, excellent biocompatibility, and low cost, CDs have great potential for application in optoelectronics, metal ion detection, and biomedicine.^20−25^

Over the past decade, people have developed a variety of techniques for preparing CDs, including physical and chemical methods. According to the relationship between the carbon source and products, these methods can be divided into two types “top-down” and “bottom-up”.^26^ The “top-down” approaches are to reduce the size of large carbon materials using chemical or physical cutting, such as laser ablation, chemical oxidation, and electrochemical decomposition, often involving complex reactions or time-consuming purification processes.^27−30^ In the “bottom-up” methods, the CDs are synthesized via appropriate molecular precursors under specific conditions such as combustion, hydrothermal, thermal pyrolysis, and ultrasonic irradiation.^31−33^ Obviously, the “bottom-up” approaches are more advantageous in terms of the requirements of reaction materials and conditions.^34^ Traditional methods for preparing doped CDs typically require a single step to functionalize and passivate the surface, which is a cumbersome process.^35,36^ In recent years, some common organic molecules, including citric acid,^37^ glucose, polyethylene glycol, urea, and so on, have been used to directly synthesize doped CQDs upon simple solid-phase pyrolysis. However, the limitations of this method are they easily get over-carbonized and agglomerated, some byproducts may be produced in pyrolysis, the size of the product is not well distributed, and it takes a long time to separate the product. Hydrothermal, microwave, or ultrasound approaches are promising to solve the above problems. For example, Cao et al.^38^ synthesized nitrogen-doped CQDs by hydrothermal treatment of citric acid and urea for effectively inhibiting the corrosion of carbon steel. Holá et al.^39^ prepared full-color fluorescent CDs by simple solvothermal decomposition in formamide using urea and citric acid as raw materials, confirming that N-doped CDs are useful for regulating CD emission. Using fumaronitrile as a precursor, Moon et al.^40^ successfully synthesized homogeneous N-GQDs using a hydrothermal method. Choi et al.^41^ synthesized highly fluorescent and amphiphilic CQDs by microwave-assisted pyrolysis of citric acid and 4,7,10-trioxa-1,13-tridecanediamine, which functioned as the A_3_ and B_2_ polyamidation-type monomer set. Some researchers have also attempted to obtain uniform CQDs with the help of microwaves or ultrasound.^42,43^

Inspired by these studies, l-glutamic acid, a non-toxic, pollution-free, readily available, and low-cost material, was used as a new precursor containing C and N to fabricate CQDs via an ultrasound-assisted hydrothermal method. Two different morphological and structured N-doped CQDs were synthesized via a one-step ultrasonic-assisted hydrothermal method at 230 °C (N-CQDs-1) and 250 °C (N-CQDs-2). Different methods were employed to characterize the features of the CQDs, including the morphology, size, structure, surface chemistry, PL properties, QY, photobleaching resistance, and influences of pH and temperature. The results indicate that N-CQDs-1 are composed of amorphous carbon with a large amount of pyroglutamic acid on their surfaces, which is reported for the first time, and N-CQDs-2 are composed of pure amorphous carbon. The fluorescence lifetimes were measured, the radiative and non-radiative decay constants were calculated, and the PL mechanism of the CQDs was qualitatively explained. Finally, the N-CQDs were used in cell imaging, and good results were obtained.

## 2 Results and Discussion

### 2.1 Morphologies of CQDs

**Figure 1:**
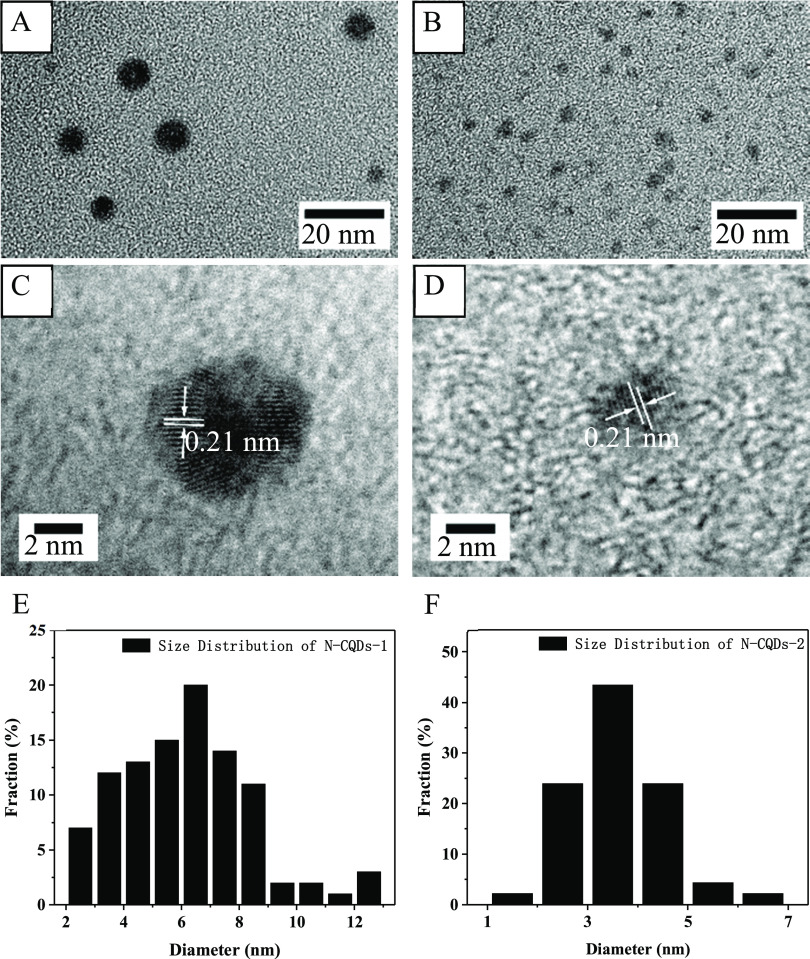
HRTEM images and size distributions of (A,C,E) N-CQDs-1 and (B,D,F) N-CQDs-2.

**Figure 2:**
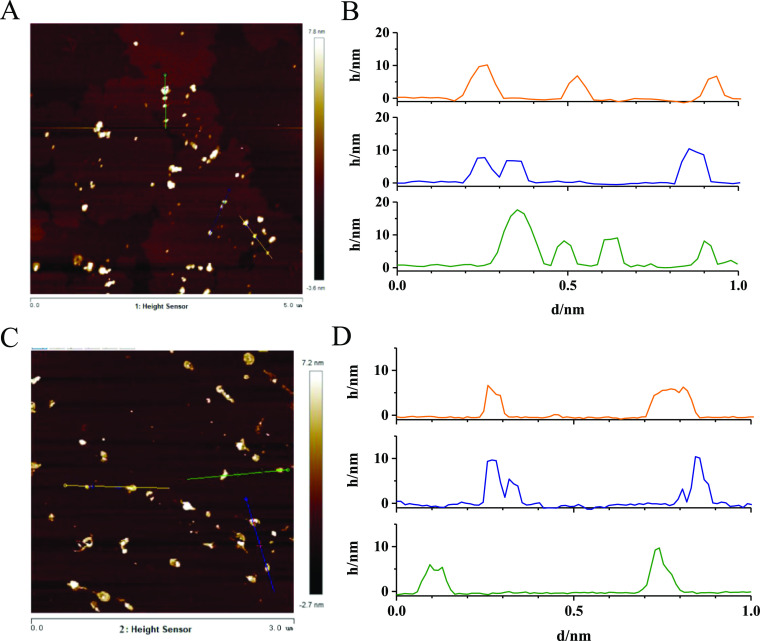
AFM images and the height distribution of (A,B) N-CQDs-1 and (C,D) N-CQDs-2.

**Figure 3:**
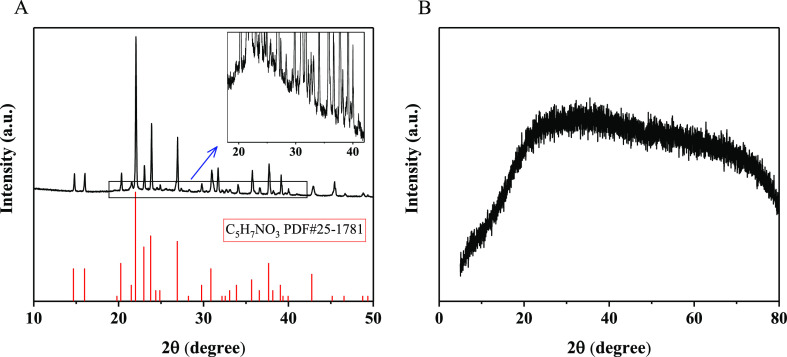
XRD patterns of (A) N-CQDs-1 and (B) N-CQDs-2. In (A), the black curve is the experimental result and the red curve corresponds to the standard sample of pyroglutamic acid; the inset shows an enlarged view of the rectangular section.

**Figure 4:**
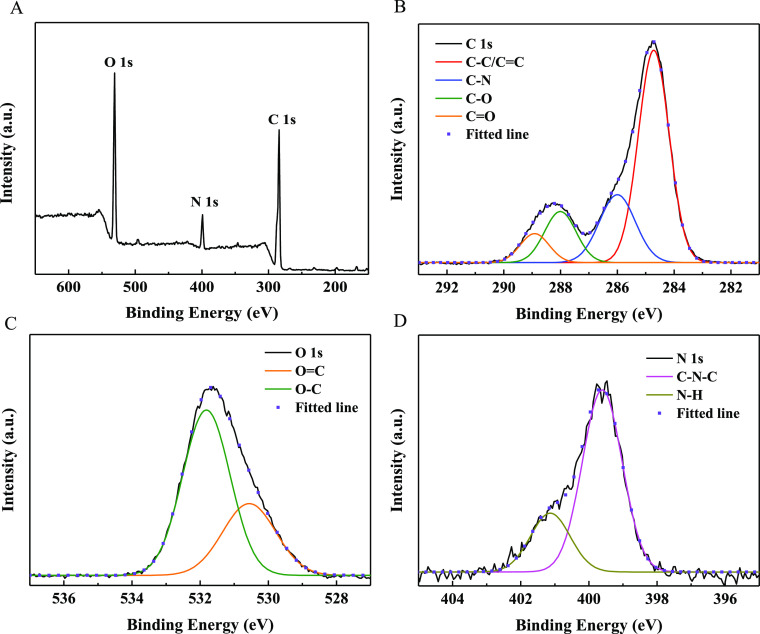
XPS spectra of N-CQDs-1: (A) full, (B) C 1s, (C) O 1s, and (D) N 1s.

**Figure 5:**
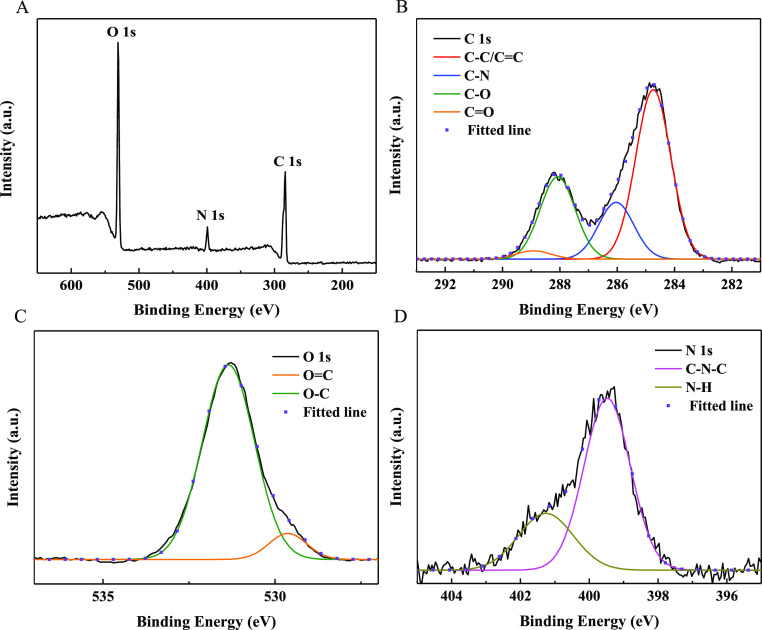
XPS spectra of N-CQDs-2: (A) full, (B) C 1s, (C) O 1s, and (D) N 1s.

**Figure 6:**
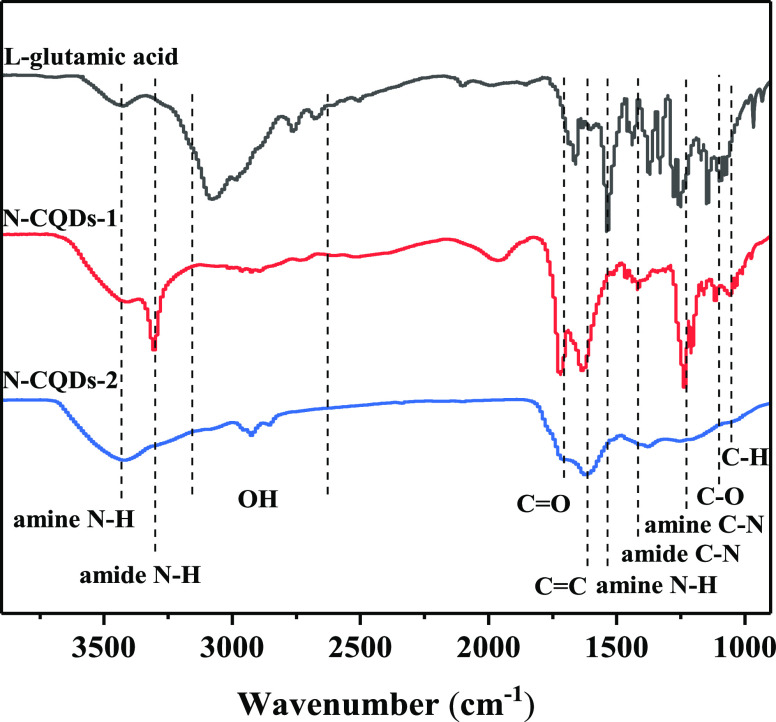
FTIR spectra of l-glutamic acid, N-CQDs-1, and N-CQDs-2.

**Figure 7:**
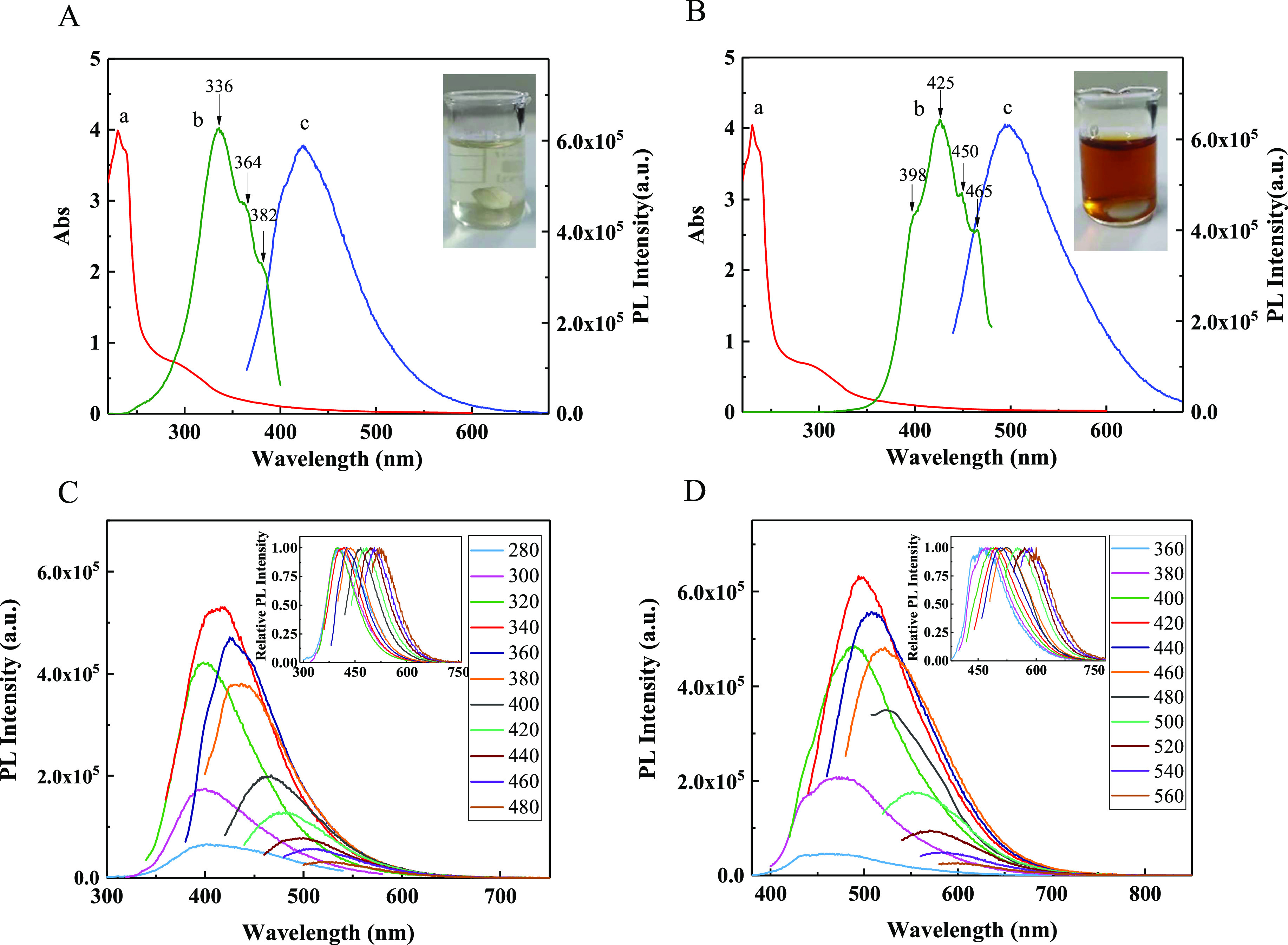
(a) Absorption, (b) excitation, and (c) emission spectra of (A) N-CQDs-1 and (B) N-CQDs-2, and the insets show the images of aqueous solutions of the two CQDs in daylight. The PL spectra of (C) N-CQDs-1 and (D) N-CQDs-2 under different excitation wavelengths.

The high-resolution transmission electron microscopy (HRTEM) results indicate that N-CQDs-1 (Figure 1A) and N-CQDs-2 (Figure 1B) are spherical nanoparticles and nanosheets, respectively, exhibiting a clear lattice, and a lattice spacing of 0.21 nm (Figure 1C,D) similar to the reported lattice spacing of carbon-based QDs prepared using different methods may reflect the (100) facet of graphite.^44−46^ The size distribution of the two CQDs is in the range of 2–13 nm (N-CQDs-1, Figure 1E) and 1–7 nm (N-CQDs-2, Figure 1F), and the average sizes are 6.2 and 3.5 nm, respectively. Atomic force microscopy (AFM) images of the CQDs (Figure 2) reveal that the thicknesses of N-CQDs-1 and N-CQDs-2 are not greater than 11.4 and 10.0 nm. According to the HRTEM and AFM results, the size of N-CQDs-1 is larger than that of N-CQDs-2.

### 2.2 Structures and Surface Chemistry of CQDs

To confirm the structures of the CQDs, powder X-ray diffraction (XRD) experiments were performed. As shown in Figure 3A, N-CQDs-1 obviously contain the crystal structure of pyroglutamic acid; however, the intensity ratios of the peak at 22° and other peaks are much higher than that of the pyroglutamic acid standard sample, which implies that in addition to pyroglutamic acid, N-CQDs-1 might contain other composites; after enlarging the rectangular area of the experimental curve (see the inset), a broad diffraction peak near 2θ = 22° can be clearly seen; combined with the results of HRTEM (Figure 1A,C), the broad diffraction peak is attributed to the amorphous carbon structure,^47,48^ so N-CQDs-1 are composed of pyroglutamic acid and amorphous carbon; and a large amount of pyroglutamic acid was found to be distributed on the surface of the amorphous carbon core. The XRD pattern of N-CQDs-2 is different from that of N-CQDs-1, and the broad peak at 2θ = 22° indicates that N-CQDs-2 have an amorphous carbon structure. Compared to the direct pyrolysis of solid-state l-glutamic acid to obtain GQDs,^47^ the ultrasonic-assisted hydrothermal approach can be potentially used to control the morphologies and structures of the CQDs.

In the fabrication process, oxygen- and nitrogen-containing functional groups may be introduced. To study the surface chemistry of the CQDs, X-ray photoelectron spectroscopy (XPS) and Fourier-transform infrared (FTIR) spectroscopy were carried out. The three representative peaks at 284.7, 531.6, and 399.6 eV in the XPS full spectrum of N-CQDs-1 (Figure 4A) correspond to C 1s, O 1s, and N 1s, respectively; the high-resolution XPS spectrum of C 1s (Figure 4B) exhibits four peaks at 284.7, 286.0, 288.0, and 288.9 eV, corresponding to the C=C/C–C, C–N, C–O/C–O–C, and C=O groups, respectively;^49,50^ the O 1s spectrum of N-CQDs-1 (Figure 4C) presents two peaks at 530.6 and 531.8 eV assigned to the C=O and the C–OH/C–O–C groups;^51,52^ and the N 1s spectrum of N-CQDs-1 (Figure 4D) depicts two peaks at 399.6 and 401.1 eV, attributed to C–N–C and N–H, respectively.^38,53^ The full spectrum of N-CQDs-2 presented in Figure 5A is similar to that of N-CQDs-1, showing three typical peaks: C 1s (284.8 eV), N 1s (399.5 eV), and O 1s (531.3 eV); the high-resolution XPS spectrum of C 1s (Figure 5B) indicates that C=C/C–C (284.7 eV), C–N (286.0 eV), C–O/C–O–C (288.0 eV), and C=O (288.9 eV) groups exist in N-CQDs-2; the O 1s spectrum (Figure 5C) of N-CQDs-2 shows the presence of C=O (529.6 eV) and C–OH/C–O–C (531.3 eV) groups in N-CQDs-2; and the high-resolution N 1s spectra (Figure 5D) of N-CQDs-2 shows that N exists in the form of C–N–C (399.5 eV) and N–H (401.2 eV) in N-CQDs-2.^38,49−53^ Comparing the XPS results of the two CQDs, most of the functional groups are similar. The above results indicate that N has been successfully doped into the structure of CDs, and the CQDs were coated with oxygen-containing functional groups, such as carboxylic, hydroxyl, carbonyl, and epoxy groups, which facilitate their hydrophilicity and dispersity in water. Because the nitrogen-containing CQDs are convenient for surface modification and they easily combine with organisms, the N-CQDs are promising for application in bio-imaging, biomarking, and optoelectronics.

The relative percentages of atomic and chemical bonds in the CQDs are different, as shown in Table 1, and the contents of C, N, and O for N-CQDs-1 and N-CQDs-2 are 64.8, 9.0, and 26.2 and 55.5, 7.4, and 37.1%, respectively. The elemental ratio of C, N, and O for l-glutamic acid is 5:1:4. Compared to the raw material, the carbon relative percentages in the two CQDs increase and those of oxygen and nitrogen decrease, indicating that the carbonization occurred during the fabrication processing. The oxygen content in N-CQDs-2 is significantly higher than that in N-CQDs-1, and the ratios of C/N of both CQDs are very close, implying that higher temperature led to more oxidation for the sample. The decrease of the nitrogen content in N-CQDs-2 was mainly caused by the increase of the oxygen content. The chemical bond ratio of C=O to C–O in N-CQDs-2 is much lower than that in N-CQDs-1, indicating that more C=O became C–O as the temperature increases, thus leading to an increase in the oxygen content.

**Table 1:** Percentages of C, N, and O and the Chemical Bonds in the Two CQDs

		peak position (eV) and relative percentage			
element and functional group		N-CQDs-1		N-CQDs-2	
C	C=C/C–C	284.7	284.7, 57.3%	284.8	284.7, 53.5%
	C–N	64.8%	286.0, 21.3%	55.5%	286.0, 17.9%
	C–O/C–O–C		287.8, 14.1%		287.8, 26.0%
	C=O		288.9, 7.3%		288.9, 2.6%
O	O=C	531.6	530.6, 32.8%	531.3	529.6, 8.9%
	O–C	26.2%	532.9, 67.2%	37.1%	531.3, 91.1%
N	C–N–C	399.6	399.6, 75.6%	399.5	399.5, 71.9%
	N–H	9.0%	401.1, 24.4%	7.4%	401.2, 28.1%

Figure 6 shows the FTIR spectra of the two CQDs, and for comparing the structural changes of the raw materials during the formation of the CQDs, the FTIR spectrum of l-glutamic acid was also measured. Comparing the three FTIR spectra shown in Figure 6, the C=C stretching of graphite resulted in a peak at 1620 cm^–1^, indicating the formation of a graphene structure after the hydrothermal reaction.^54,55^ The wide peak in the range of 2600–3200 cm^–1^ is derived from the O–H extension of carboxylic acids.^47,56^ At the same time, the tensile bonding of carboxylic acid C=O and amide C=O in CQDs formed a strong peak at 1600–1750 cm^–1^.^57^ Compared to raw materials, the weakened O–H and the elongated C=O peaks for CQDs indicate l-glutamic acid underwent decarboxylation in the formation of CQDs, and the majority of C=O was attributed to the amide C=O and the minority of C=O was ascribed to the carboxylic acid C=O. The stretching vibrations of amine N–H (at about 3400 cm^–1^) in three FTIR spectra indicate the presence of amine groups in the raw material, N-CQDs-1 and N-CQDs-2.^58^ In addition, absorptions at 3300, 1420, 1230, and 1140 cm^–1^ were strengthened, indicating the existence of amide N–H, amide C–N, amine C–N, and C–O bands in CQDs, respectively.^47,59−61^ The peaks of amide N–H (3300 cm^–1^) and amine C–N (1230 cm^–1^) in the N-CQDs-1 are obviously stronger than those in the N-CQDs-2, implying that the content of N is more in the N-CQDs-1, which coincides well with the XPS results. The amine N–H stretching band at 1530 cm^–1^ in CQDs is obviously weakened, illustrating more amines in l-glutamic acid were changed into amides in CQDs. The C–H stretching bands obviously weakened at 1130–1064 cm^–1^, illustrating the depolymerization and decomposition of the l-glutamic acid during carbonization.^62^ The above results show that oxygen-containing functional groups have been formed on the surface of CQDs, and N has been successfully doped into the CQDs, which is consistent with the results of XPS.

### 2.3 Optical Properties and the PL Mechanism of CQDs

Optical properties are significant for the practical application of CQDs. Using ultraviolet–visible (UV–vis) absorption and PL spectroscopy techniques, the optical–physical properties of the CQDs were investigated. The obtained UV–vis absorption spectra and PL spectra are shown in Figure 7. The insets in Figure 7A,B show the aqueous solution images of N-CQDs-1 and N-CQDs-2 in daylight, exhibiting light yellow and orange yellow.

N-CQDs-1 shows obvious absorption peaks at 230 and 298 nm (Figure 7A,a); the absorption peaks of N-CQDs-2 (Figure 7B,a) are similar to those of N-CQDs-1, except that the peak at 298 nm is slightly lower. As the oxygen- or nitrogen-containing functional groups are located at the surface of the CQDs, the related surface states may be induced between the π band (highest occupied molecular orbital) and the π* band (lowest unoccupied molecular orbital). Generally, the absorption peaks at 230 and 298 nm are attributed to the electronic transitions of π → π* of C=C and n → π* of the C=O bond, respectively.^63^ Compared with the 282 nm absorption peak in ref (64), the absorption peak at 298 nm of the CQDs has a red shift, which may be caused by N-doped (C=N/C–N).^65^

**Scheme 1:**
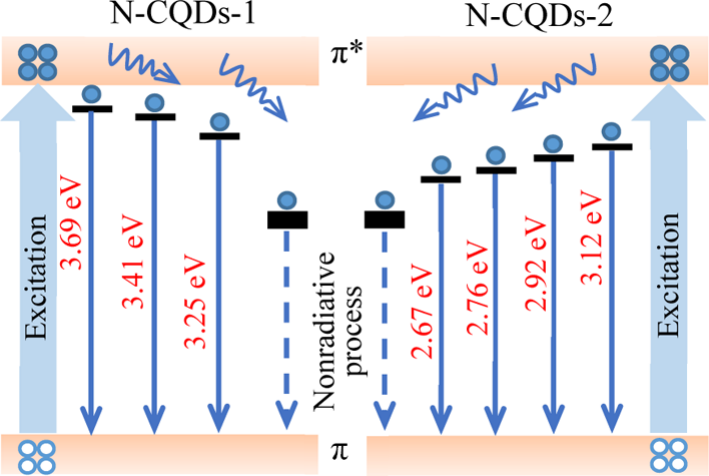
Proposed Energy Band Diagram of the CQDs

It is generally believed that the luminescence properties of CQDs are caused by π-conjugated domains determined by the carbon core and the surface state determined by hybridization of the carbon backbone and the connecting chemical groups.^63^ To investigate the recombination events responsible for the PL emission, the excitation spectra of N-CQDs-1 (Figure 7A,b, λ_em_ = 424 nm) and N-CQDs-2 (Figure 7B,b, λ_em_ = 500 nm) were obtained, three (336, 364, and 382 nm) and four excitation peaks (398, 425, 450, and 465 nm) were observed for N-CQDs-1 and N-CQDs-2, respectively. As depicted in Scheme 1, three electronic transitions of N-CQDs-1 at 336 nm (3.69 eV), 364 nm (3.41 eV), and 382 nm (3.25 eV) can be regarded as three types of the transitions; and four electronic transitions for N-CQDs-2 at 398 nm (3.12 eV), 425 nm (2.92 eV), 450 nm (2.76 eV), and 465 nm (2.67 eV) can be considered to the four types of the transitions.^66,67^ The energy band diagram of the two CQDs is proposed as shown in Scheme 1. The excited electrons at the π* band may emit photons directly or relax to the surface states, these relaxed electrons may emit photons by radiative combination or not emit photons because of non-radiative combination, thus the PL spectra of the CQDs may exhibit excitation-dependent properties for the distributed surface states. The experimental results proved this guess. The emission peaks of N-CQDs-1 shift from 340 to 540 nm upon changing the excitation wavelengths from 280 to 480 nm (Figure 7C); and the emission peaks of N-CQDs-2 move from 420 to 620 nm when varying the excitation wavelengths in the range of 340–560 nm (Figure 7D); the excitation-dependent PL spectra of the two CQDs are similar to some published results.^66,68−70^ N-CQDs-2 emits a longer wavelength fluorescence, the main reason is N-CQDs-2 possesses a higher oxygen content than N-CQDs-1 (XPS results, Table 1), which is consistent with ref (70). The PL of the two CQDs is strong enough to be observed by the naked eye, implying its good application prospect in fluorescence imaging. Under excitation at 340 and 420 nm, respectively, the N-CQDs-1 and N-CQDs-2 showed the strongest PL peaks at 424 nm (Figure 7A,c) and 500 nm (Figure 7B,c), and the PL spectra exhibit mirror symmetry to the excitation spectra.

To further explore the fluorescence features of the developed CQDs, we measured their fluorescence QYs and lifetimes at room temperature and neutral pH. The obtained QY values of N-CQDs-1 and N-CQDs-2 under excitation at 340 and 420 nm, respectively, are up to 40.5% (quinine sulfate as a standard substance) and 13.2% (rhodamine B as a standard reference dye). Higher C/O ratios (2.47 for N-CQDs-1 and 1.50 for N-CQDs-2) and higher N doping percentages (9.0% for N-CQDs-1 and 7.4% for N-CQDs-2) are the two plausible reasons that might lead to the QY of N-CQDs-1 that is far more than that of N-CQDs-2.

**Figure 8:**
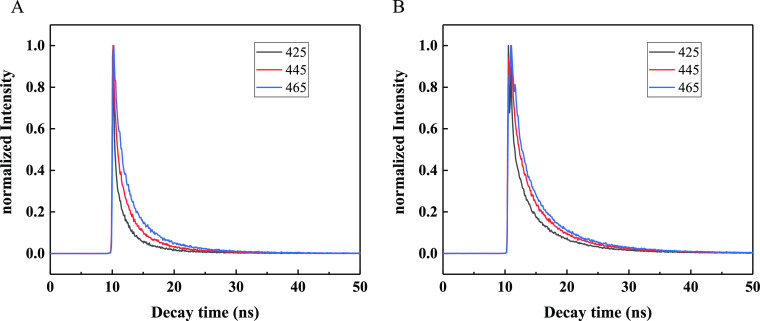
Time-resolved PL decay curves of (A) N-CQDs-1 and (B) N-CQDs-2. λ_em_ = 425, 445, and 465 nm; λ_ex_ = 375 nm.

The fluorescence lifetimes of the CQDs were analyzed using the time-correlated single photon counting (TCSPC) technique at different emission wavelengths with 375 nm excitation. As shown in Figure 8, all the PL decay curves can be best fitted with a biexponential function, where they exhibited short and long fluorescence lifetime components; these components stem from direct radiative emission from the surface and relaxation from the core to the surface states, respectively.^71^ For N-CQDs-1, the contribution percentage of short components to fluorescence is close to 50%, while for N-CQDs-2, it is close to 40%, indicating that the two CQD structures are indeed different, which may be due to the increase of core carbonization caused by the increase of the synthesis temperature. The lifetimes of the CQDs at 425, 445, and 465 nm are summarized in Table 2. The average lifetimes (Av·s) of N-CQDs-1 and N-CQDs-2 are in the range of 4.75–6.21 and 6.77–8.04 ns, which are in good agreement with those of CQDs grown by pyrolysis and hydrothermal methods.^54,72^ Comparing the two CQDs, the size of N-CQDs-2 is smaller than that of N-CQDs-1 (Figure 1), but the fluorescence lifetime is longer, which is consistent with ref (73). As shown in Table 2 and Figure 8, when the λ_em_ increases from 425 to 465 nm, the average lifetimes of the N-CQDs-1(N-CQDs-2) increase from 4.75 (6.77) ns to 6.21 (8.04) ns. The shorter λ_em_ leads to a shorter lifetime. The corresponding increase of the long component is more than that of the short component, which indicates that the surface states of the two CQDs play a major role in luminescence. Additionally, the radiative (*K*_r_) and non-radiative (*K*_nr_) decay rate constants can be obtained from the measured QYs and PL lifetimes using^40^
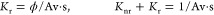
1*K*_r_ and *K*_nr_ values of N-CQDs-1 are 8.53/7.49/6.52 × 10^7^ s^–1^ and 12.52/10.99/9.58 × 10^7^ s^–1^, and *K*_r_ and *K*_nr_ values of the N-CQDs-2 are 1.95/1.77/1.64 × 10^7^ s^–1^ and 12.82/11.65/10.80 × 10^7^ s^–1^, respectively; the two CQDs possess almost the same magnitude of *K*_nr_; however, the *K*_r_ values of N-CQDs-1 are about 5–6 times higher than those of N-CQDs-2. For the shorter lifetime or larger radiative decay rate constant, N-CQDs-1 has a higher QY than N-CQDs-2. The nanosecond lifetimes of the CQDs demonstrate their perspective applications in optoelectronics and bio-imaging.

**Table 2:** Fluorescence Lifetimes of CQDs at Different Emission Wavelengths with 375 nm Excitation

	Em (nm)	A1 (%)	s1 (ns)	A2 (%)	s2 (ns)	Av·s (ns)	χ^2^
N-CQDs-1	425	46.59	1.1411	53.41	5.4117	4.75	1.337
	445	48.26	1.4792	51.74	6.2761	5.41	1.267
	465	50.81	1.8893	49.19	7.3529	6.21	1.161
N-CQDs-2	425	37.42	1.7352	62.58	7.4729	6.77	2.915
	445	40.45	2.2259	59.55	8.3961	7.45	2.533
	465	42.90	2.4377	57.10	9.1585	8.04	2.317

### 2.4 Stability of CQDs

**Figure 9:**
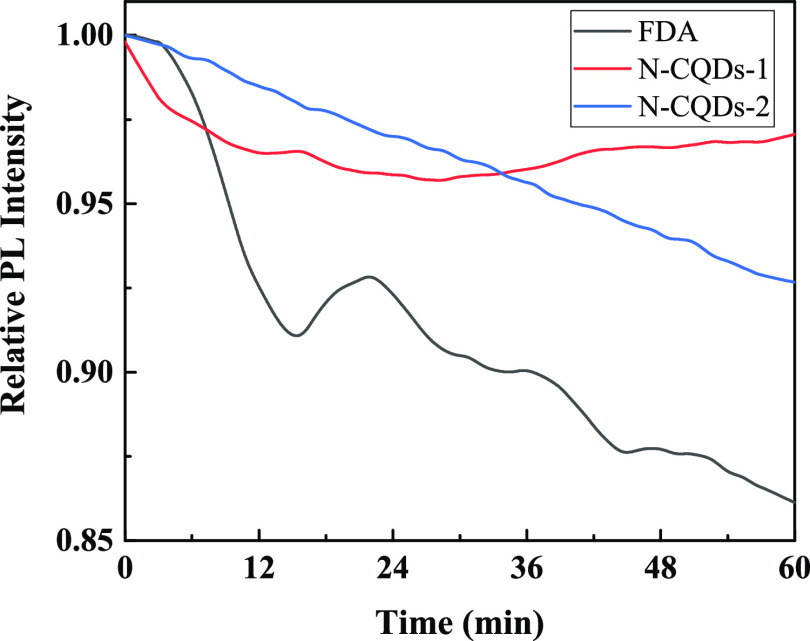
Photobleaching resistance curves of CQDs and the traditional organic dye of FDA.

Many factors, such as the pH, temperature, and preservation time, can influence the optical properties of CQDs. For practical applications, the photostability of the CQDs is very important. In this section, the effects of photobleaching resistance, pH, and temperature on the photostability of the CQDs were probed. Compared to the traditional organic dyes, such as fluorescein diacetate (FDA), the CQDs show excellent photobleaching resistance. As shown in Figure 9, the PL intensity of FDA was bleached by 14% under ultraviolet light (350 nm) irradiation for 1 h, however, the photobleaching ratios of N-CQDs-1 and N-CQDs-2 are only 4 and 8% respectively within the same irradiation period, which are far less than those of FDA, implying that CQDs were not easily aggregated into dimers or photodecomposed into small particles. This superior photostability provides CQDs with great potential for in vitro and in vivo fluorescence imaging applications.

**Figure 10:**
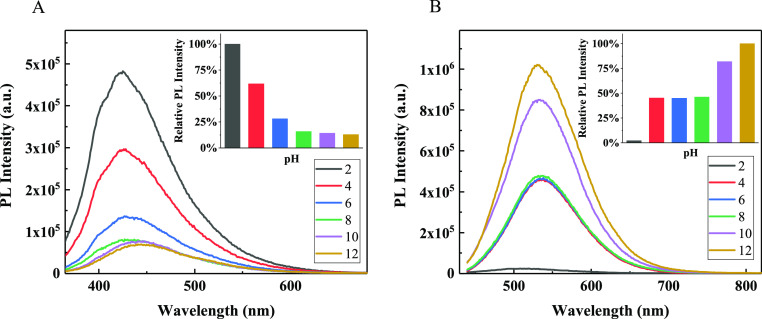
PL spectra of (A) N-CQDs-1 and (B) N-CQDs-2 at varying pH. The inset shows the corresponding bar charts.

**Figure 11:**
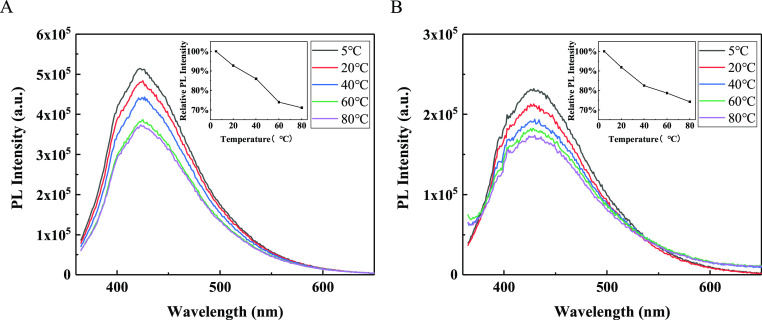
PL spectra of (A) N-CQDs-1 and (B) N-CQDs-2 at various temperatures. The insets show the peak intensities at different temperatures.

The experimental results of the PL spectra of CQDs obtained by varying the pH values in the range of 2–12 are illustrated in Figure 10. The fluorescence intensities of N-CQDs-1 (Figure 10A) sharply decrease at the pH value in the range of 2–6; however, there is no obvious intensity change of the PL spectra in the pH range of 8–12 and the PL intensity at pH = 2 is 5 times higher than that at pH = 12; and in an alkaline environment, N-CQDs-1 exhibits excellent photostability but the QY has to be sacrificed slightly. For N-CQDs-2, the tendency of PL intensities with pH values is opposite to N-CQDs-1, the stronger the alkalinity, the higher the PL intensities; within the pH value of 4–8, the PL intensities exhibit a slight change. Thus, the two CQDs are also suitable for use in a neutral environment. The PL intensities of the two CQDs decrease with the increase of the temperature in the range of 5–80 °C (Figure 11); the insets in Figure 11A,B are the normalized PL intensities; the PL intensities of N-CQDs-1 and N-CQDs-2 at 80 °C are 70% higher than those at 5 °C and 90% at 25 °C; thus, at room temperature (20–30 °C), the PL spectra of the two CQDs are slightly influenced by the environmental temperature. Therefore, it can be inferred that the two CQDs are stable at room temperature.

### 2.5 Fluorescence Imaging of BV2 Cells Using CQDs

**Figure 12:**
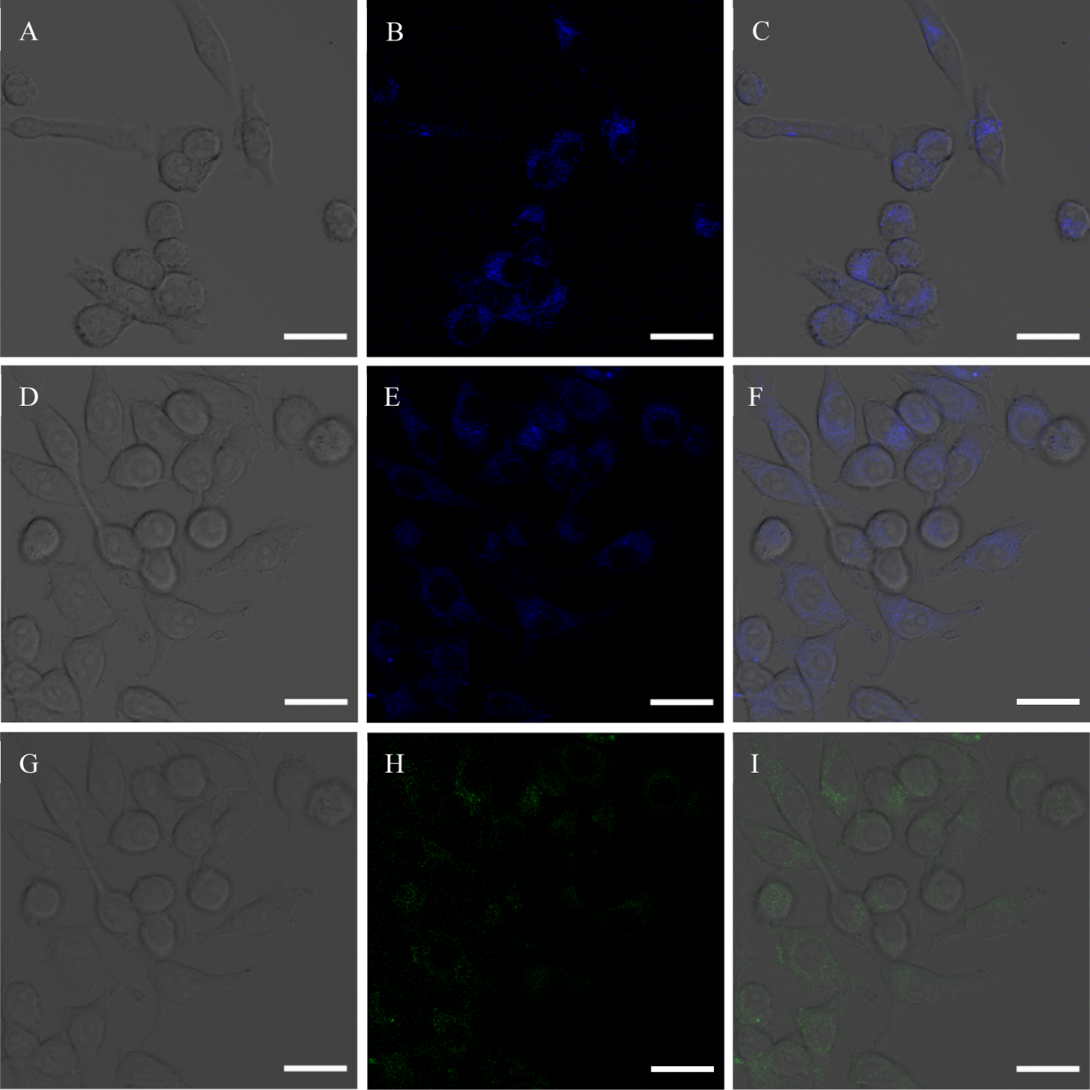
In vitro cell imaging. (A–C) and (D–F) Confocal images of BV2 cell addition of N-CQDs-1 and N-CQDs-2 in (A,D) bright field, (B,E) blue channel (excitation: 402.7 nm; emission: 425–475 nm), and (C,F) overlay channel, respectively. (G–I) Confocal images of BV2 cell addition of N-CQDs-2 in (G) bright field, (H) green channel (excitation: 488.2 nm; emission: 500–550 nm), and (I) overlay channel. All images have a scale of 25 μm.

Based on the above excellent fluorescence properties of the prepared CQDs, the in vitro fluorescence imaging using the developed CQDs was explored at different excitation wavelengths. After the CQDs were incubated with BV2 cells in PBS for 1 h, the fluorescence images of the cells were obtained using a confocal laser microscope as illustrated in Figure 12; Figure 12A,D shows the microimages of the BV2 cells in the bright field; Figure 12B,E shows the microimages of the BV2 cells incubated with N-CQDs-1 and N-CQDs-2, respectively, upon excitation at 405 nm; BV2 showed a strong blue (LP450) color, and the CQDs could be seen to be evenly distributed in the cytoplasm and rarely entered the nucleus; and Figure 12C,F shows the overlays. Under excitation at 488 nm, only the BV2 cells incubated with N-CQDs-2 exhibited a bright green color, as is shown in Figure 12H. Thus, N-CQDs-1 can be used for in vitro fluorescence imaging in the blue color range, and N-CQDs-2 is suitable for imaging in blue and green color ranges. The strong fluorescence of the BV2 cells incubated with CQDs suggests that CQDs could penetrate the cells and could mark the nucleus and the cytoplasm of BV2 cells.^74−76^ The uptake of CQDs by the BV2 cells would be similar to the pathway for nanoparticles, which is endocytosis.

## 3 Conclusions

In summary, using a natural amino acid (l-glutamic acid) as a precursor, two different morphological and structured N-doped CQDs were synthesized via a one-step ultrasonic hydrothermal method at 230 °C (N-CQDs-1) and 250 °C (N-CQDs-2). The XRD results indicate that N-CQDs-1 are the QDs composed of amorphous carbon with a large amount of pyroglutamate on their surfaces, which are reported for the first time, while N-CQDs-2 are composed of pure amorphous carbon. The nitrogen contents in N-CQDs-1 and N-CQDs-2 are 9.0 and 7.4%, respectively. The PL spectra of the two CQDs are all excitation-dependent because the surface states dominate the emission. N-CQDs-1 and N-CQDs-2 emit strong blue and blue-green fluorescence. The fluorescence QY of N-CQDs-1 (40.5%) is much higher than that of N-CQDs-2 (13.2%) because the former possesses a shorter fluorescence lifetime and a larger radiative decay constant *K*_r_. The strong acidic and strong alkaline environments have an obvious influence on the PL intensities, but in the pH range of 6–8, the PL spectra of the CQDs are basically stable. The two CQDs exhibited excellent photobleaching resistance and good temperature stability at room temperature and neutral pH (within 6 months). The results of BV2 cell imaging using the CQDs are good. Our results show that the ultrasonic-assisted hydrothermal method is a facile approach to control the morphologies and structures of the CQDs, which are promising for bioimaging and optoelectronic applications.

## 4 Experimental Section

### 4.1 Materials

l-Glutamic acid was purchased from Alfa Aesar (USA). Deionized water (18.3 MU cm) was produced from a Millipore water purification system. Dulbecco’s modified Eagle medium (DMEM) and fetal bovine serum (FBS) were bought from Gibco (USA). Penicillin (100 U/mL) and streptomycin sulfate (100 U/mL) were bought from Invitrogen (USA).

### 4.2 CQD Synthesis

**Scheme 2:**
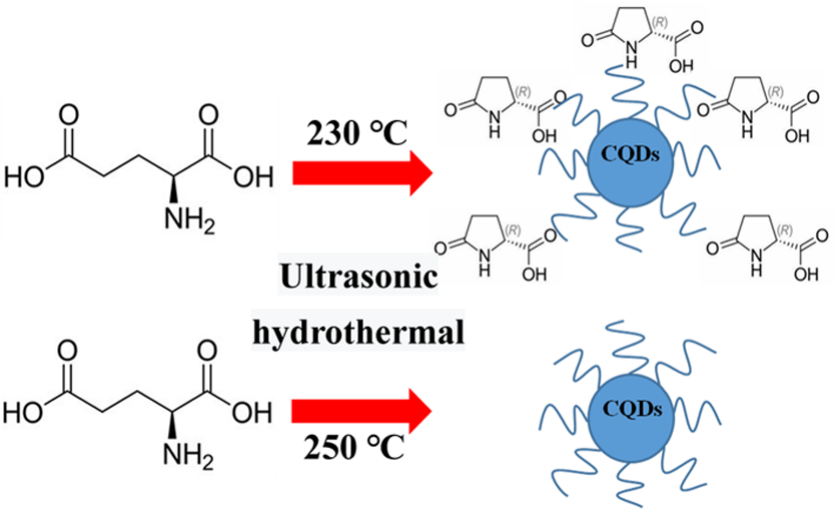
Schematic Diagram of the Preparation of CQDs by l-Glutamic Acid

Typically, 60 mL l-glutamic acid aqueous (0.45 M) was added into the reaction kettle connected to the transducer of an ultrasonic generator. During the reaction, the ultrasonic generator was kept at 50% power. The solution was heated up to a certain temperature; after keeping the temperature constant for 4 h, the solution was cooled down to room temperature; and the final solution changed into yellow and no obvious precipitation was observed, which indicates the CQDs were produced. Via a filter with a 0.22 μm hole size and 24 h dialysis (1 KD), a pure CQDs solution was obtained. After freeze-drying, solid CQDs were obtained. Two CQDs were synthesized at 230 and 250 °C, respectively, and named N-CQDs-1 and N-CQDs-2. The formation of CQDs from l-glutamic acid is illustrated in Scheme 2.

### 4.3 Characterization

A high-resolution transmission electron microscope (JEOL JEM-2100F, Japan) was used to characterize the morphology and lattice of the CQDs. The size distribution of the CQDs was analyzed using ImageJ software. The surface morphology of the CQDs was observed using an atomic force microscope (Seiko-SPA400, Japan). The UV–vis absorption spectra of the CQDs were obtained by a UV–vis absorption spectrophotometer (HITACHI UH5300, Japan). The PL spectroscopy was performed using a FluoroMax-4 fluorescent spectrometer (Horiba JY, USA). FTIR spectra were obtained using a Nicolet iN10 FTIR spectrometer (Thermo Fisher Scientific, USA) with a resolution of 4 cm^–1^ in the range of 4000 to 500 cm^–1^. XPS (Thermo ESCALAB 250Xi, Thermo Fisher, USA) was used to analyze the relative contents of carbon, nitrogen, and oxygen and chemical bonds in the CQDs. The crystalline structures of the CQDs were characterized using an XRD setup (Bruker D8 ADVANCE, BRUKER AXS, Germany) with Cu Kα radiation (λ = 1.5406 Å). The fluorescence lifetimes of the CQDs were detected using TCSPC with a LED (375 nm) equipped on a time-resolved fluorescence spectrometer (Edinburgh F900, UK), the emission wavelengths of the CQDs are at 425, 445, and 465 nm. An incubator (Thermo Fisher Scientific, MA, USA) was employed to culture BV2 cells. The cell imaging was carried out using a confocal laser scanning microscope (Nikon A1R HD25, Japan).

### 4.4 Detection of QY

Rhodamine B (65%) and quinine sulfate (54%) were used as standard substances.^36,77^ The QY was calculated according to the following equation,
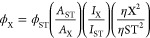
2where the subscripts ST and X denote the standard and the under studied sample, respectively; ϕ is the QY; *I* represents the integrated area of the PL spectrum; *A* is the absorbance at the excited wavelength; and η is the refractive index of the solution. The refractive indices for the standard and the sample are all 1.33. The solutions N-CQDs-1 and N-CQDs-2 were excited at 340 and 420 nm, respectively.

### 4.5 In Vitro Fluorescence Imaging of BV2 Cells

The fluorescence imaging of BV2 cells treated with the CQDs was conducted at various wavelengths. Briefly, the BV2 murine microglial cell line was cultured in DMEM containing 1% penicillin–streptomycin and 10% FBS in an incubator with 5% CO_2_ and 95% humidity at 37 °C. The culture solution was changed every other day. BV2 cells were placed on a confocal plate with a density of 5 × 10^4^ cells/mL. When the cell density reached about 80%, 200 μg/mL CQDs was added to the cell medium and cultured at 37 °C and 5% CO_2_ for 1 h.^78^ Finally, washing the BV2 cells three times by using PBS (pH 7.4), the morphology of the BV2 cell was observed and imaged via a confocal laser scanning microscope.
